# Prevalence and severity of urinary incontinence and associated factors in Iranian postmenopausal women: a cross-sectional study

**DOI:** 10.1186/s12894-023-01186-w

**Published:** 2023-02-13

**Authors:** Afsaneh Alizadeh, Maryam Montazeri, Fatemeh Shabani, Soheila Bani, Shirin Hassanpour, Mahsan Nabighadim, Mojgan Mirghafourvand

**Affiliations:** 1 Department of Midwifery, Faculty of Nursing and Midwifery, Tabriz University of Medical Sciences, Tabriz, Iran; 2 Physical Medicine and Rehabilitation Research Center, Tabriz University of Medical Sciences, Tabriz, Iran; 3 Women’s Reproductive Health Research Center, Tabriz University of Medical Sciences, Tabriz, Iran; 4 Department of Medicine, Faculty of Medicine, Ardabil University of Medical Sciences, Ardabil, Iran; 5 Social Determinants of Health Research Center, Faculty of Nursing and Midwifery, Tabriz University of Medical Sciences, Tabriz, Iran

**Keywords:** Stress urinary incontinence, Urgency urinary incontinence, Mixed urinary incontinence, Menopause

## Abstract

**Background:**

Urinary incontinence (UI) is one of the most common symptoms during menopause, leading to a decreased quality of life and limited social activities. This study aimed to determine the prevalence and severity of urinary incontinence and associated risk factors in postmenopausal women.

**Methods:**

It was a cross-sectional study using cluster sampling on 433 postmenopausal women in Tabriz-Iran, 2021–2022. Data were collected using questionnaires of socio-demographic characteristics, Questionnaire for Urinary Incontinence Diagnosis (QUID), and International Consultation on Incontinence Questionnaire-Urinary Incontinence Short Form (ICIQ-UISF). Multivariate logistic regression was used to determine factors related to urinary incontinence.

**Results:**

The overall prevalence of urinary incontinence was 39.5%; 20.6% stress urinary incontinence (SUI), 10.4% urgency urinary incontinence (UUI), and 8.5% mixed urinary incontinence (MUI). Multivariate logistic regression analysis showed that the prevalence of SUI (aOR 0.38; 95% CI 0.18–0.77) and UUI (aOR 0.38; 95% CI 0.15–0.94) was significantly lower in women with three childbirths than the ones with fewer childbirths. Also, the odds of UUI increased significantly in women at the 50–55 age range (aOR 3.88; 95% CI 1.16–12.93) than those less than 50 years.

**Conclusion:**

Due to the high prevalence of urinary incontinence in postmenopausal women, caregivers should screen for early diagnosis and appropriate treatment of urinary incontinence to prevent its destructive impact on the quality of life.

## Background

Menopausal transition is a biological situation associated with reproductive function loss and various health problems [1]. Menopause, the permanent cessation of menstruation, results from the loss of ovarian follicular activity [2]; it occurs between 45 and 55 years of age. Women spend about one-third of their lives in the postmenopausal period [3]. Menopausal symptoms include physical and vasomotor symptoms (hot flushes and night sweats), osteoporosis, urinary tract atrophy and infections, urinary incontinence (UI), increased risk of cardiovascular diseases, decreased libido, and sexual dysfunction [4].

The prevalence of UI during menopause varies from 9 to 69% [5]. The overall rate is 46% in Iranian women, indicating the urgent need to pay attention to this issue [6]. UI is the complaint of any unintentional passing of urine [7]. It can be classified as follows: (A) Stress incontinence; unintentional and sudden loss of urine caused by increased intra-abdominal pressure with activities such as laughing, pushing, sneezing, and coughing, (B) Urgency incontinence; a sudden, intense urge to urinate followed by an involuntary loss of urine, and (C) Mixed incontinence; a combination of stress and urgency incontinence [8]. The frequency and severity of symptoms are related to age, body mass index (BMI), drowsiness, and constipation [9].

Urinary incontinence is not a life-threatening disease; however, it can affect the social, psychological, familial, occupational, physical, and sexual aspects of patients' lives [10]. UI leads to a decrease in the quality of life, creating social isolation and limiting social activities [11]. It is associated with decreased sexual desire and sexual satisfaction, also the feeling of shame and stress [12]. According to recent data, the peak prevalence of UI is at the age of menopause [13]. Estrogen and collagen deficiency result in decreased elasticity of the pelvic floor, atrophic changes, and urinary incontinence [14]. Advanced age, usually coinciding with menopause, also has debilitating effects on the pelvic organs and tissues [15].

Due to the limited research on the Iranian population [16, 17], the increase in mean life expectancy, and the prevalence of urinary incontinence and its impact on various aspects of life, our study aimed to determine the prevalence and severity of urinary incontinence and its related factors in a health centers-based sample of postmenopausal women in Tabriz-Iran. Caregivers better provide appropriate treatments to reduce the prevalence of UI through educating, preventing, and reducing the impact of risk factors.

## Methods

### Type of study, setting and participants

It was a cross-sectional and health center-based study on a population of 433 postmenopausal women in Tabriz-Iran 2021–2022. The whole people in Iran have access to free healthcare services, answerable to the Ministry of Health and Medical Education. All health protocols were observed during the Coronavirus epidemic.

Inclusion criteria were no menses for one year, the ability to speak and listen, and having a personal health record at the health center of Tabriz. Exclusion criteria were mental and motor disabilities.

The sample size was calculated to be 289 people based on a previous study by Tashakori et al. [18] and the estimation formula of a proportion (*P* = 25.1%, α = 0.05, d = 0.05). We selected 433 ones, considering the design effect of 1.5 due to cluster sampling.

### Sampling

**Fig. 1:**
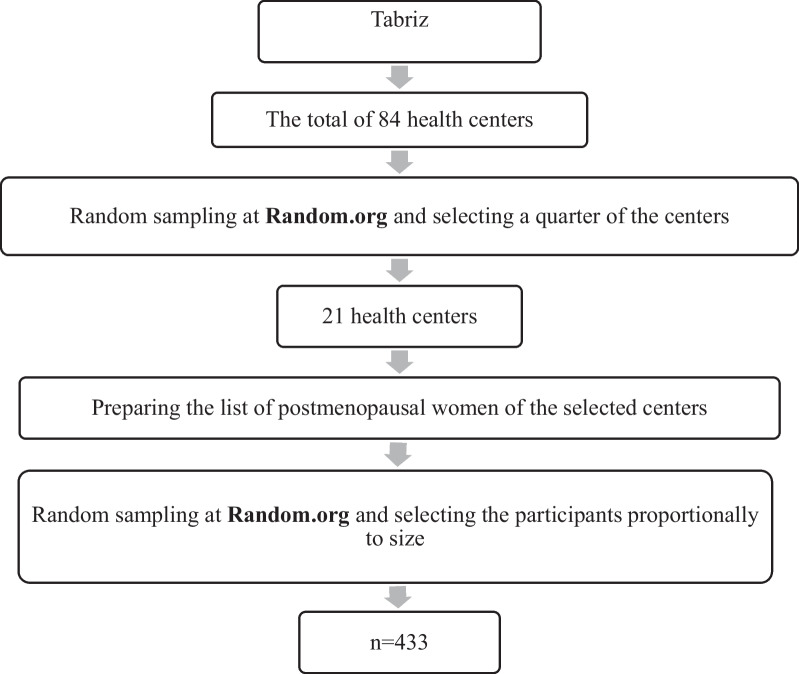
Sampling frame

The study began after obtaining the written approval of the Ethics Committee of Tabriz University of Medical Sciences (Ethical code: IR.TBZMED.REC.1400.494). The participants were selected from the centers by cluster sampling method. Tabriz city has 84 health centers; using the website: www.random.org, a quarter of the health centers were randomly selected. The list of postmenopausal women was accessed through integrated health system (SIB) files. The number of selected samples from each center was determined proportionally based on the sample size and the number of postmenopausal women covered by each center. They were randomly selected from the prepared list. The researcher contacted them and explained the research aims and objectives. Considering the inclusion/exclusion criteria, women willing to participate in the study attended a meeting at the health center. After comprehensively explaining the project, they were asked to read and sign a consent form. Socio-demographic characteristics, Questionnaire for Urinary Incontinence Diagnosis (QUID), and International Consultation on Incontinence Questionnaire-Urinary Incontinence Short Form (ICIQ- UISF) were completed via interviews by the researchers (first and second authors). Due to the Coronavirus epidemic and social distancing, the interviews were conducted individually in an air-conditioned room of health centers. We assessed a sample of 433 postmenopausal women (Fig. 1).

### Data collection tools

Questionnaires of socio-demographic characteristics, QUID, and ICIQ-UISF were used to collect data.

### Socio-demographics characteristics questionnaire

The items were age, menopause age, menopause duration, parity, hormone replacement therapy, education, job, home status, sufficiency of income for living costs, and exercise. The face and content validity of the questionnaire were evaluated qualitatively by Tabriz University of Medical Sciences members.

### Questionnaire for urinary incontinence diagnosis (QUID)

It is a self-administered, 6-item questionnaire to distinguish between SUI and UUI. The first three items focus on the symptoms of stress incontinence; the other ones focus on urgency incontinence. A score of 4 and above in the first three items indicates stress incontinence and a score of 6 and above in the second three items is the criterion for urgency incontinence; the existence of both cases indicates the mixed type. Based on the 5-point Likert scale, the scores are: never (0), rarely (1), sometimes (2), often (3), most of the time (4), and always (5). This tool has been validated for the Iranian population [19].

### International consultation on incontinence questionnaire-urinary incontinence short form (ICIQ-UISF)

This questionnaire contains six items, examining the individual’s condition in the last four weeks. The items are about demographic characteristics (1–2), frequency of UI (3), amount of leakage (4), and the impact on the quality of life (5); items 3–5 represent the actual score. The sixth item is related to the time and type of urine leakage (not considered). The total score ranges from 0 to 21; mild (1–5), moderate (6–12), severe (13–18), and very severe (19–21). A higher score indicates the intensity of UI. The validity and reliability of this tool were confirmed in 2012 by Haj Ebrahimi et al. in Iran [20].

### Statistical analysis

All data were analyzed using SPSS-24 software. Descriptive statistics, frequency (percentage) and mean (standard deviation) were used to describe socio-demographic characteristics and types of urinary incontinence. The Chi-square test was used to determine the association between UI with socio-demographic characteristics; variables related to the types of urinary incontinence entered the model with *p* < 0.2. Multivariate logistic regression was used to determine the predictors of UI. The results were presented as the adjusted odds ratio (aOR) with the 95% confidence interval (95% CI). A p-value less than 0.05 was considered significant.

## Results

### Description of socio-demographic characteristics

The mean (SD) age of participants was 54.5 (2.2). About two-thirds of women (67.7%) had given birth to less than four children and stated a menopause age before 50 years (64.7%). Most of them had less than eight years since their menopause (80.8%) and did not use hormone therapy to reduce menopausal symptoms (98.2%). Majority of them (81.1%) had an education level less than a diploma. Most women were housewives (88.0%), living in their private homes (94.0%). Most of them (92.7%) stated that household income was relatively/completely sufficient for living expenses. Two thirds of women (62.1%) stated that they do not exercise during the day (Table 1).

**Table 1:** Socio-demographic characteristics among participants (*n* = 433)

Variables	N (%)	Variables	N (%)
*Age (Years)*		*Education*	
≤ 50	25(5.8)	Illiterate	87 (20.1)
51–55	245(56.06)	Primary school	154 (35.6)
≥ 56	163(37.6)	Secondary school	74 (17.1)
Mean (SD)	54.5 (2.2)	High school	36 (8.3)
*Parity*		Diploma	55 (12.7)
≤ 2	149(34.4)	University	27 (6.2)
3	144 (33.3)	*Job*	
≥ 4	69 (15.9)	Homemaker	381 (88.00)
*Menopause age*		Employed	52 (12.0)
≤ 40	42 (9.7)	*Home*	
46–50	238 (55.0)	Personal	407(94.0)
≥ 51	153 (35.3)	Rental	26(6.0)
Mean (SD)	49.6 (2.5)	*Income*	
*Menopause duration*		Completely sufficient	32 (7.4)
≤ 3	158 (36.5)	Relatively sufficient	270 (62.4)
4–7	192 (44.3)	Insufficient	131 (30.3)
≥ 8	83 (19.2)	*Doing exercise*	
Mean (SD)	4.8 (2.8)	No	269 (62.1)
*Hormone replacement therapy*		Yes	164 (37.9)
No	425 (98.2)		
Yes	8 (1.8)		

### Prevalence of urinary incontinence types

The overall prevalence of urinary incontinence was 39.5%; 20.6% stress urinary incontinence, 10.4% urgency urinary incontinence, and 8.5% mixed urinary incontinence. Urinary incontinence was mild in 81.3% of cases, 12.2% moderate, 5.8% severe, and 0.7% very severe (Table 2).

**Table 2:** Prevalence of types of incontinence and its severity

	Stress urinary incontinence	Urgent urinary incontinence	Mixed urinary incontinence
Yes (N %)	89 (20.6)	45 (10.4)	37 (8.5)
No (N %)	344 (79.4)	388 (89.6)	396 (91.5)

Severity of incontinence			
Mild (N %)	Moderate (N %)	Severe (N %)	Very Severe (N %)
352 (81.3)	53 (12.2)	25 (5.8)	3 (0.7)

### Relationship between socio-demographic characteristics and urinary incontinence types based on bivariate and multivariate analyses

Based on the chi-square test, there was a statistically significant relationship between the variables of parity (*P* = 0.004), education (*P* = 0.002), and job (*P* = 0.021) with stress urinary incontinence. The highest frequency of SUI was in women with a parity of 4 and more (44.9%), secondary/lower education level (85.4%), and housewives (95.5%). There was a statistically significant relationship between parity and urgency urinary incontinence (*P* = 0.035); the highest frequency of UUI was in women with a parity of 4 and more (48.9%).

There was a statistically significant relationship between the variables of parity (*P* = 0.013), education (*P* = 0.001) and income (*P* = 0.023) with mixed urinary incontinence. The highest frequency of MUI was in women with a parity of 4 and more (54.1%), secondary/lower education level (94.6%), and insufficient income (45.9%) (Table 3).

**Table 3:** Relationship between socio-demographic characteristics and types of urinary incontinence based on the Chi-squared test

Variables	Stress urinary incontinence			Urgent urinary incontinence			Mixed urinary incontinence		
	N (%)			N (%)			N (%)		
	Yes	No	*P* value	Yes	No	*P* value	Yes	No	*P* value
*Age (Years)*			0.128			0.165			0.799
≤ 50	9 (10.1)	16 (4.7)		5 (11.1)	20 (50.2)		3 (8.1)	22 (5.6)	
51–55	50 (56.2)	195 (56.7)		27 (60)	218 (56.2)		21 (56.8)	224 (56.6)	
≥ 56	30 (33.7)	133 (38.7)		13 (28.9)	150 (3807)		13 (35.1)	150 (27.9)	
*Menopause age*			0.096			0.575			0.839
≤ 45	14 (15.7)	28 (8.1)		4 (8.9)	38 (9.8)		3 (8.1)	39 (9.8)	
46–50	45 (50.6)	193 (56.1)		28 (62.2)	210 (54.1)		22 (59.5)	216 (54.5)	
≥ 51	30 (33.7)	123 (35.8)		13 (28.9)	140 (36.1)		12 (32.4)	141 (35.6)	
*Menopause duration*			0.649			0.178			0.367
≤ 3	36 (40.4)	122 (35.5)		18 (40.0)	140 (36.1)		16 (43.2)	142 (35.9)	
4–7	36 (40.4)	156 (45.3)		23 (51.1)	169 (43.6)		17 (45.9)	175 (44.2)	
≥ 8	17 (19.1)	66 (19.2)		4 (8.9)	79 (20.4)		4 (10.8)	79 (19.9)	
*Parity*			0.004			0.035			0.013
≤ 2	19 (21.3)	130 (37.8)		10 (22.2)	139 (35.8)		7 (18.9)	142 (35.9)	
3	30 (33.7)	114 (33.1)		13 (28.9)	131 (33.8)		10 (27.0)	134 (33.8)	
≥ 4	40 (44.9)	100 (29.1)		22 (48.9)	118 (30.4)		20 (54.1)	120 (30.3)	
*Education*			0.002			0.076			0.001
Secondary and Lower education	76 (85.4)	239 (69.5)		38 (84.4)	277 (71.4)		35 (94.6)	280 (70.7)	
High school & beyond	13 (14.6)	105 (30.5)		7 (15.6)	111 (28.6)		2 (5.4)	116 (29.3)	
*Job*			0.021			0.396			0.051
Homeworker	85 (95.5)	296 (86.5)		42 (93.3)	339 (87.4)		36 (97.3)	345 (87.1)	
Employed	4 (4.4)	48 (13.9)		3 (6.6)	49 (12.6)		1 (2.7)	51 (12.9)	
*Hormone replacement therapy*			0.370			1.000			1.000
No	89 (100)	336 (97.7)		45(100)	380(97.9)		37 (100)	388 (98.0)	
Yes	0 (0.0)	8 (2.3)		0(0.0)	8(2.1)		0 (0.0)	8 (2.0)	
*Income*			0.332			0.064			0.023
Completely sufficient	4 (4.5)	28 (8.1)		1(2.2)	31(8.0)		1 (2.7)	31 (7.8)	
Relatively sufficient	56 (62.9)	214 (62.6)		26(57.8)	244 (62.9)		19 (51.4)	251 (63.4)	
Insufficient	29 (32.6)	102 (29.7)		18(40.0)	113 (29.1)		17 (45.9)	114 (28.8)	
*Doing exercise*			0.507			0.189			0.079
No	58 (65.2)	211 (61.3)		32(71.1)	237 (61.1)		28 (75.7)	241 (60.9)	
Yes	31 (34.8)	133 (38.7)		13(28.9)	151 (38.9)		9 (24.3)	155 (39.1)	
*Home*			0.863			0.502			0.151
Personal	84 (94.4)	323 (93.9)		44(97.8)	363 (93.6)		37 (100)	370 (93.4)	
Rental	21 (6.1)	5 (5.6)		1(2.2)	25 (6.4)		0 (0.0)	26 (6.6)	

Multivariate logistic regression analysis showed that the prevalence of SUI (aOR 0.38; 95% CI 0.18–0.77) and UUI (aOR 0.38; 95% CI 0.15–0.94) was significantly lower in women with three childbirths than the ones with two childbirths or lower. Also, the odds of UUI increased significantly in women at the 50–55 age range (aOR 3.88; 95% CI 1.16–12.93) than those less than 50 years (Table 4).

**Table 4:** Predictors of incontinence types through logistic regression test

Variables	Stress urinary incontinence		Urgent urinary incontinence		Mixed urinary incontinence	
	N (%)		N (%)		N (%)	
	OR (95% CI)	*P* value	OR (95% CI)	*P* value	OR (95% CI)	*P* value
*Age (Years)*						
≤ 50	1		1			
51–55	1.80 (0.55–.80)	0.326	3.88 (1.16–12.93)	0.027		
≥ 56	1.12 (0.60–2.10)	0.706	1.78 (0.85–3.72)	0.126		
*Menopause age*						
≤ 45	1					
46–50	3.05 (0.93–10.00)	0.065				
≥ 51	1.17 (0.62–2.21)	0.625				
*Menopause duration*						
≤ 3	1					
4–7	2.16 (0.74–6.34)	0.158				
≥ 8	1.46 (0.63–3.39)	0.373				
*Parity*						
≤ 2	1		1		1	
3	0.38 (0.18–0.77)	0.008	0.38 (0.15–0.94)	0.037	0.54 (0.21–1.42)	0.216
≥ 4	0.59 (0.33–1.06)	0.081	0.47 (0.21–1.01)	0.055	0.54 (0.24–1.23)	0.146
*Education*						
Secondary and Lower education	1		1		1	
High school & beyond	1.49 (0.70–3.15)	0.292	1.16 (0.43–3.14)	0.762	4.14 (0.80–21.29)	0.088
*Job*						
Homemaker	1				1	
Employed	2.07 (0.66–6.46)	0.209			2.19 (0.24–19.90)	0.485
*Income*						
Completely sufficient			1			
Relatively sufficient			0.36 (0.04–3.28)	0.370	1.04 (0.10–10.80)	0.971
Insufficient			0.78 (0.39–1.53)	0.476	0.61 (0.30–1.24)	0.176
*Doing exercise*						
No			1			
Yes			1.21 (0.59–2.47)	0.603	1.45 (0.64–3.26)	0.362

## Discussion

The exact prevalence of urinary incontinence, especially the different types, has not been assessed in Iran. A similar study was conducted in another city (Yazd) in this country [17]; however, it didn’t categorize the types of UI. Our study was the first that assessed the prevalence and severity of UI with its types in the Iranian population. In the present study, the overall prevalence of urinary incontinence was 39.5%; 20.6% SUI, 10.4% UUI, and 8.5% MUI. The odds of UI increased with age and decreased in women with three childbirths than the ones with two childbirths or lower.

About two in five postmenopausal women had urinary incontinence in this study. In a meta-analysis by Batmani et al. (2021), the prevalence of UI in older women was reported as 37.1% in the world, the highest one in Asia (45.1%) [21]. The prevalence estimates for UI ranged from 25.1 to 26.4% in women aged 45–64 in the Netherlands [22], 35% in premenopausal women aged 45–55 in Australia [23], 38% in women aged 60–84 in the US [24], 46% in women with the mean age of 48 in different cities of Iran [6], and 54% in Yazd-Iran [17]. The difference observed in the results may be due to racial and ethnic differences between countries and participants' socio-demographic characteristics. Age and parity are among the main factors after race and ethnicity.

In the present study, the prevalence urinary incontinence was 20.6% for SUI (the most common type), 10.4% for UUI, and 8.5% for MUI. In a study by Doley et al. (2008), among American women who reported incontinence, 49.8% was for SUI, 34.3% for MUI, and 15.9% for UUI [25]. In another study by Minassian et al. (2008), the overall prevalence of UI was 49.2%; 23.7% SUI, 14.5% MUI, and 9.9% UUI. SUI peaked at the fifth decade [26]. In the study of Zhu et al. (2009) in Beijing, the prevalence of UI in postmenopausal women was 30.9%. The estimated prevalence of SUI, UUI, and MUI was 18.9%, 2.6%, and 9.4%, respectively [27]. In a study by Ajith et al. (2019) in India, the prevalence of UI in postmenopausal women was 26.47%; 13.9% SUI, 7.2% MUI, and 5.4% UUI [28]. All these studies are in line with the present one; SUI was the most common type of urinary incontinence. Parity, one of the main factors affecting urinary incontinence, causes SUI more than other types [29]; it can be the reason for the difference in the prevalence of types of urinary incontinence.

In this study, the prevalence of UUI increased significantly with age. In the study by Bardsley et al. (2016), UI was a multifactorial condition associated with age; it was more common in women than men in all age groups [30]. In a study by Hunskaar et al., the prevalence of UI varied between 5 and 69% and increased with aging [31]. Several studies have defined age as one of the main risk factors of UI [32, 33]. The severity of anterior, apical, and posterior vaginal wall prolapse increases with age [34]. Pelvic floor disorders are associated with age-related connective tissue and neuromuscular changes; other factors include obesity, pulmonary diseases, and diabetes which are common in older people [35]. Due to the increased life expectancy after menopause, age is one of the main factors affecting the prevalence of UI and determining the quality of life [36].

In this study, the prevalence of SUI and UUI decreased significantly in women with three childbirths than the ones with two childbirths or lower. In the study of Garcia et al., the frequency of UI in parous women was significantly higher than in nulliparous ones [37]. Zhu et al. stated that vaginal delivery increases the risk of SUI [38]. Also, Senturk et al. (2012) reported vaginal delivery as the main risk factor for UI [39]. In a cross-sectional study, the effect of the number of deliveries varied from 8% in nulliparous to 17% in primiparous ones. Parity was correlated with UI; the first delivery was the most significant [40]. In the recent research by Larsudd et al., the first vaginal delivery contributed the highest increase in the risk of SUI surgery (threefold); the second vaginal delivery contributed the lowest increase in this risk (∼1/10 of the first vaginal delivery) [41]. In another study by Karmarkar et al., the urethral sphincter was smaller and the bladder neck position was lower in parous women than in nulliparous ones. However, these differences were not progressive with increasing parity [42]. Vaginal delivery is associated with the risk of damage to pelvic floor muscles, nerves, and connective tissue [43]. The mentioned studies agree on the effect of first delivery on the prevalence of UI, but whether this prevalence increases with increasing parity is still controversial.

## Strengths and limitations

The use of standard and valid questionnaires and cluster sampling are the strengths of this study. The data was collected in a completely accurate process without any missing. One of the limitations was due to the Coronavirus epidemic and social distancing; this problem was solved by conducting individual interviews and providing an air-conditioned room. Considering the nature of cross-sectional studies, the relationships found between urinary incontinence with socio-demographic characteristics do not accurately indicate a causal relationship. Also, the associated risk factors could not be demonstrated in this study due to the inadequate sample size.

## Conclusion

According to the results, more than one-third of the postmenopausal women were suffering from urinary incontinence. The odds of urinary incontinence increased with age and decreased in women with three childbirths than the ones with two childbirths or lower. Due to the increased longevity after menopause, appropriate treatments should be considered to improve the quality of life of postmenopausal women. We recommend conducting a longitudinal study with long-term follow-ups and various treatment methods to find the best one.

## Data Availability

The datasets generated and/or analyzed during the current study are not publicly available due to limitations of ethical approval involving the patient data and anonymity but are available from the corresponding author on reasonable request.

## Abbreviations

QUID: Questionnaire for Urinary Incontinence Diagnosis
ICIQ-UISF: International Consultation on Incontinence Questionnaire-Urinary Incontinence Short Form
UI: Urinary Incontinence
SUI: Stress Urinary Incontinence
UUI: Urgency Urinary Incontinence
MUI: Mixed Urinary Incontinence
aOR: Adjusted Odds Ratio
95% CI: 95% Confidence Interval
